# Supplementary material for: Real-World Incidence of Immune-Related Adverse Events Associated with Nivolumab Plus Ipilimumab in Patients with Advanced Renal Cell Carcinoma: A Retrospective Observational Study
Source: J Clin Med. 2021 Oct 18;10(20):4767. doi: 10.3390/jcm10204767 (PMC8541457; doi:10.3390/jcm10204767)
Supplement: Supplementary file 1 [file jcm-10-04767-s001.zip › jcm-1373216-supplementary.pdf]

Table S1. Adverse events in CTCAE ver5 assessed in this study.

	Adverse events	Grade 1	Grade 2	Grade 3	Grade 4	Grade 5
Endocrine disorders	Adrenal insufficiency	Asymptomatic; clinical or diagnostic observations only; intervention not indicated	Moderate symptoms; medical intervention indicated	Severe symptoms; hospitalization indicated	Life-threatening consequences; urgent intervention indicated	Death
	Hypophysitis	Asymptomatic or mild symptoms; clinical or diagnostic observations only; intervention not indicated	Moderate; minimal, local or noninvasive intervention indicated; limiting age-appropriate instrumental ADL	Severe or medically significant but not immediately life-threatening; hospitalization or prolongation of existing hospitalization indicated; limiting self care ADL	Life-threatening consequences; urgent intervention indicated	Death
	Hypopituitarism	Asymptomatic or mild symptoms; clinical or diagnostic observations only; intervention not indicated	Moderate; minimal, local or noninvasive intervention indicated; limiting age-appropriate instrumental ADL	Severe or medically significant but not immediately life-threatening; hospitalization or prolongation of existing hospitalization indicated; limiting self care ADL	Life-threatening consequences; urgent intervention indicated	Death
	Hyperthyroidism	Asymptomatic; clinical or diagnostic observations only; intervention not indicated	Symptomatic; thyroid suppression therapy indicated; limiting instrumental ADL	Severe symptoms; limiting self care ADL; hospitalization indicated	Life-threatening consequences; urgent intervention indicated	Death
	Hypothyroidism	Asymptomatic; clinical or diagnostic observations only; intervention not indicated	Symptomatic; thyroid replacement indicated; limiting instrumental ADL	Severe symptoms; limiting self care ADL; hospitalization indicated	Life-threatening consequences; urgent intervention indicated	Death
	Hyperglycemia	Abnormal glucose above baseline with no medical intervention	Change in daily management from baseline for a diabetic; oral antiglycemic agent initiated; workup for diabetes	Insulin therapy initiated; hospitalization indicated	Life-threatening consequences; urgent intervention indicated	Death
Cardiac disorders	Myocarditis	(-)	Symptoms with moderate activity or exertion	Severe with symptoms at rest or with minimal activity or exertion;	Life-threatening consequences; urgent intervention indicated (e.g.,	Death

				intervention indicated; new onset of symptoms	continuous IV therapy or mechanical hemodynamic support)	
Nervous system disorders	Radiculitis	Mild symptoms	Moderate symptoms; medical intervention indicated; limiting instrumental ADL	Severe symptoms; limiting self care ADL	Life-threatening consequences; urgent intervention indicated	Death
Renal and urinary disorders	Creatinine increased	>ULN - 1.5 x ULN	>1.5 - 3.0 x baseline; >1.5 - 3.0 x ULN	>3.0 x baseline; >3.0 - 6.0 x ULN	>6.0 x ULN	(-)
	Acute kidney injury	(-)	(-)	Hospitalization indicated	Life-threatening consequences; dialysis indicated	Death
	Proteinuria	1+ proteinuria; urinary protein ≥ULN - <1.0 g/24 hrs	Adult: 2+ and 3+ proteinuria; urinary protein 1.0 - <3.5 g/24 hrs; Pediatric: Urine P/C (Protein/Creatinine) ratio 0.5 - 1.9	Adult: Urinary protein >=3.5 g/24 hrs; 4+ proteinuria; Pediatric: Urine P/C (Protein/Creatinine) ratio >1.9	(-)	(-)
Respiratory disorders	Pneumonitis	Asymptomatic; clinical or diagnostic observations only; intervention not indicated	Symptomatic; medical intervention indicated; limiting instrumental ADL	Severe symptoms; limiting self care ADL; oxygen indicated	Life-threatening respiratory compromise; urgent intervention indicated (e.g., tracheotomy or intubation)	Death
Skin disorders	Rash maculo-papular	Macules/papules covering <10% BSA with or without symptoms (e.g., pruritus, burning, tightness)	Macules/papules covering 10 - 30% BSA with or without symptoms (e.g., pruritus, burning, tightness); limiting instrumental ADL; rash covering > 30% BSA with or without mild symptoms	Macules/papules covering >30% BSA with moderate or severe symptoms; limiting self care ADL	(-)	(-)
	Pruritus	Mild or localized; topical intervention indicated	Widespread and intermittent; skin changes from scratching (e.g., edema, papulation, excoriations, lichenification, oozing/crusts); oral intervention indicated; limiting	Widespread and constant; limiting self care ADL or sleep; systemic corticosteroid or immunosuppressive therapy indicated	(-)	(-)

			instrumental ADL			
	Skin hypopigmentation	Hypopigmentation or depigmentation covering <10% BSA; no psychosocial impact	Hypopigmentation or depigmentation covering >10% BSA; associated psychosocial impact	(-)	(-)	(-)
Gastrointestinal	Colitis	Asymptomatic; clinical or diagnostic observations only; intervention not indicated	Abdominal pain; mucus or blood in stool	Severe abdominal pain; peritoneal signs	Life-threatening consequences; urgent intervention indicated	Death
Hepatobiliary	Alkaline phosphatase increased	>ULN - 2.5 x ULN if baseline was normal; 2.0 - 2.5 x baseline if baseline was abnormal	>2.5 - 5.0 x ULN if baseline was normal; >2.5 - 5.0 x baseline if baseline was abnormal	>5.0 - 20.0 x ULN if baseline was normal; >5.0 - 20.0 x baseline if baseline was abnormal	>20.0 x ULN if baseline was normal; >20.0 x baseline if baseline was abnormal	(-)
	Hepatic failure	(-)	(-)	Asterixis; mild encephalopathy; drug-induced liver injury (DILI); limiting self care ADL	Life-threatening consequences; moderate to severe encephalopathy; coma	Death
Musculoskeletal	Arthritis	Mild pain with inflammation, erythema, or joint swelling	Moderate pain associated with signs of inflammation, erythema, or joint swelling; limiting instrumental ADL	Severe pain associated with signs of inflammation, erythema, or joint swelling; irreversible joint damage; limiting self care ADL	(-)	(-)
	Myositis	Mild pain	Moderate pain associated with weakness; pain limiting instrumental ADL	Pain associated with severe weakness; limiting self care ADL	Life-threatening consequences; urgent intervention indicated	(-)

Table S2. ACTH and cortisol levels at baseline and the diagnosis of ACTH deficiency.

	Baseline		**At the onset of ACTH deficiency**	
**Case**	**ACTH, pg/mL**	**Cortisol, μg/dL**	**ACTH, pg/mL**	**Cortisol, μg/dL**
JMU1	(-)	(-)	<1.5	0.9
JMU2	21.2	9.2	1.8	<0.9
JMU4	59.3	22.1	2.4	1.6
JMU5	25.8	11.3	<1.5	1.3
SMC3	30.4	26.5	2.6	0.17
SMC10	22.3	6.14	2.5	0.88
SMC12	23	12.5	<1.5	1.62
SMC15	76.1	12.3	8	0.65
SMC17	17.5	15.3	3.4	0.8
SCC1	(-)	(-)	3.6	<1.0
SCC2	41.3	17.3	11.2	5.6

SCC6	(-)	(-)	1.6	<1.0
SCC7	(-)	(-)	1.8	0.71
SCC8	(-)	(-)	13.3	1.2
SCC9	(-)	(-)	6.7	<1.0
SCC10	(-)	(-)	11	2
SCC11	51	10.51	2	0.53
SCC14	56	10.7	3	3.38

Normal range of ACTH and cortisol: 7.2 - 63.3 pg/mL and 4.5 – 21.1 μg/dL, respectively. Low levels of values were blue-highlighted; JMU: Jichi Medical University Saitama Medical Center; SMC: Saitama Medical Center Saitama Medical University; SCC: Saitama Cancer Center; (-): not assessed.

Table S3. TSH, fT4 and fT3 levels at baseline, and the diagnosis of hyperthyroidism and primary hypothyroidism.

			**Baseline**			**At hyperthyroidism**			**At primary hypothyroidism**		
	Hyper-thyroidism, grade	Hypo-thyroidism, grade	TSH, mIU/ml	fT4, ng/dl	fT3, ng/dl	TSH, mIU/ml	fT4, ng/dl	fT3, ng/dl	TSH, mIU/ml	fT4, ng/dl	fT3, ng/dl
JMU1	1	0	0.53	1.55	2.82	0.03	1.43	2.71	(-)	(-)	(-)
JMU2	1	0	0.76	1.09	2.87	0.3	1.12	3.59	(-)	(-)	(-)
JMU5	1	2	1.03	1.24	2.41	0.06	4.05	7.09	18.02	0.92	1.92
JMU8	3	2	1.14	0.99	2.42	<0.10	>8	13.7	10.27	0.19	<0.5
SMC1	2	2	1.32	1.16	2.98	0.02	2.4	5.91	14.3	0.92	2.09
SMC6	1	2	1.14	0.89	2.47	0.02	1.79	4.2	6.04	0.54	2.08
SMC10	1	0	2.28	1.29	3.2	0.02	1.84	5.31	(-)	(-)	(-)
SMC11	0	2	8.1	1.15	2.56	(-)	(-)	(-)	154	0.36	1.32
SMC12	1	0	2.30	1.26	2.98	0.01	1.94	2.05	(-)	(-)	(-)
SMC14	1	2	2.84	1.2	2.56	0.02	5.11	7.67	26	0.68	1.77
SMC16	1	0	3.53	1.06	2.49	0.09	1.15	2.99	(-)	(-)	(-)
SMC19	1	2	3.3	1.16	2.97	0.03	51	10.7	119	0.59	1.52
SCC5	2	2	0.57	1.22	(-)	0.01	6.84	13.5	26.8	0.23	1.26
SCC6	1	0	4.08	1.19	3.14	0.02	1.77	(-)	(-)	(-)	(-)
SCC10	2	0	0.93	0.92	(-)	0.09	0.58	1.9	(-)	(-)	(-)
SCC11	2	0	1.74	1.06	(-)	0.02	1.85	3.29	(-)	(-)	(-)
SCC13	1	2	0.61	1.11	2.98	0.02	0.88	(-)	50.8	0.17	(-)
SCC14	3	0	0.29	1.42	2.92	0.01	3.2	(-)	(-)	(-)	(-)

Normal range of TSH, FT4 and FT3: 0.5 – 5 mIU/ml, 0.7 – 1.9 ng/dL and 2.3 – 4.0 ng/dL, respectively. High and low levels of values were red- and blue-highlighted, respectively; TSH: thyroid-stimulating hormone; fT4: free thyroxine; fT3: free triiodothyronine; JMU: Jichi Medical University Saitama Medical Center; SMC: Saitama Medical Center Saitama Medical University; SCC: Saitama Cancer Center; (-): not assessed.
